# Importance of uncharged polar residues and proline in the proximal two-thirds (Pro^107^–Ser^128^) of the highly conserved region of mouse ileal Na^+^-dependent bile acid transporter, Slc10a2, in transport activity and cellular expression

**DOI:** 10.1186/1472-6793-13-4

**Published:** 2013-02-04

**Authors:** Tohru Saeki, Kosuke Sato, Shiho Ito, Keisuke Ikeda, Ryuhei Kanamoto

**Affiliations:** 1 Laboratory of Molecular Nutrition, Kyoto Prefectural University, Nakaragi, Shimogamo, Sakyo-ku, Kyoto, 606-8522, Japan; 2 Current affiliation: Laboratory of Physiological Function of Food, Division of Food Science and Biotechnology, Graduate School of Agriculture, Kyoto University, Gokasho, Uji city, Kyoto Prefecture, 611-0011, Japan

**Keywords:** Bile acid, Enterohepatic circulation, Ileal sodium-dependent bile acid transporter

## Abstract

**Background:**

SLC10A2-mediated reabsorption of bile acids at the distal end of the ileum is the first step in enterohepatic circulation. Because bile acids act not only as detergents but also as signaling molecules in lipid metabolism and energy production, SLC10A2 is important as the key transporter for understanding the *in vivo* kinetics of bile acids. SLC10A family members and the homologous genes of various species share a highly conserved region corresponding to Gly^104^–Pro^142^ of SLC10A2. The functional importance of this region has not been fully elucidated.

**Results:**

To elucidate the functional importance of this region, we previously performed mutational analysis of the uncharged polar residues and proline in the distal one-third (Thr^130^–Pro^142^) of the highly conserved region in mouse Slc10a2. In this study, proline and uncharged polar residues in the remaining two-thirds of this region in mouse Slc10a2 were subjected to mutational analysis, and taurocholic acid uptake and cell surface localization were examined. Cell surface localization of Slc10a2 is necessary for bile acid absorption. Mutants in which Asp or Leu were substituted for Pro^107^ (P107N or P107L) were abundantly expressed, but their cell surface localization was impaired. The S126A mutant was completely impaired in cellular expression. The T110A and S128A mutants exhibited remarkably enhanced membrane expression. The S112A mutant was properly expressed at the cell surface but transport activity was completely lost. Replacement of Tyr^117^ with various amino acids resulted in reduced transport activity. The degree of reduction roughly depended on the van der Waals volume of the side chains.

**Conclusions:**

The functional importance of proline and uncharged polar residues in the highly conserved region of mouse Slc10a2 was determined. This information will contribute to the design of bile acid-conjugated prodrugs for efficient drug delivery or SLC10A2 inhibitors for hypercholesterolemia treatment.

## Background

Bile acids are synthesized from cholesterol in the liver and secreted into the small intestine as components of bile for the digestion and absorption of lipids and lipid-soluble vitamins. In addition to the detergent action of bile acids, which aids in the digestion and absorption of lipid and lipid-soluble nutrients by forming micelles with biliary phospholipids and cholesterol, bile acids are now appreciated as signaling molecules that control lipid metabolism and energy production [1-6]. At the distal end of the ileum, 95%–98% of bile acids are effectively reabsorbed by an ileal sodium-dependent bile acid transporter (SLC10A2, also designated ASBT, ISBT, or IBAT) and returned to the liver *via* portal circulation. Among the transporters that are expressed in the liver, intestine, and bile duct and are involved in enterohepatic circulation of bile acids, SLC10A2 is the key transporter for understanding the *in vivo* kinetics of bile acids given that reabsorption of bile acids by SLC10A2 is the first step in enterohepatic circulation. SLC10A2 is the second member of the solute carrier family 10, and consists of 348 amino acids. SLC10A2 is expressed in the ileum, cholangiocytes, and kidney, and contributes to the maintenance of the bile acid pool and cholesterol homeostasis [7-9]. Transport of bile acids by SLC10A2 is facilitated by sodium symport in an electrogenic process with a 2:1 Na^+^/bile acid stoichiometry [10]. Given that bile acids are synthesized from cholesterol, inhibition of bile acid reabsorption *via* SLC10A2 inhibition has been used as a cholesterol-lowering therapy. Moreover, due to its high transport capacity in the ileum, SLC10A2 is also an attractive target for the prodrug strategy to enhance drug bioavailability [11,12].

The membrane topology and detailed transport mechanism of SLC10A2 have been studied. Hydropathy analysis and membrane insertion scanning revealed that SLC10A2 has an extracellular N-terminus and a cytoplasmic C-terminus [13,14]. The exact membrane topology remains controversial: *in vitro* translation studies using membrane insertion scanning suggested a 9-transmembrane (TM) topology, whereas N-glycosylation scanning mutagenesis and dual-label epitope insertion scanning mutagenesis support a 7-TM topology [13-19]. The recently published crystal structure of a bacterial homolog of SLC10A2 from *Neisseria meningitidis* (designated ASBT_NM_) supports the 9-TM topology [20].

Protein regions and amino acid residues of SLC10A2 involved in membrane trafficking, substrate recognition, and substrate permeation have been identified. The cytoplasmic tail of rat Slc10a2 acts as a sorting signal for apical trafficking, and Ser^335^ and Thr^339^ phosphorylations are crucial for apical targeting [21]. Computational analysis based on homology-modeling and remote-threading techniques revealed that Asp^282^ and Leu^283^ of human SLC10A2 are involved in hydrogen bond formation with the 12α-hydroxyl group of bile acids [19]. A series of analyses using the substituted-cysteine accessibility method revealed that in the 7-TM model TM7 (Phe^287^–Tyr^308^) lines the substrate translocation pathway, TM4 (Ile^160^–Met^180^) forms part of the pathway, Asp^124^ interacts with the 7α-hydroxyl group of bile acids, and the extracellular loop (EL) 1 corresponding to Val^99^–Ser^126^ acts as a Na^+^ sensor [22-24]. Glu^261^ in EL3 has also been shown to act as a Na^+^ sensor, and EL1 and EL3 have been proposed to act as re-entrant loop segments [25,26]. Despite such extensive studies, the mechanisms underlying the binding and transport of bile acids remain unclear.

**Figure 1:**
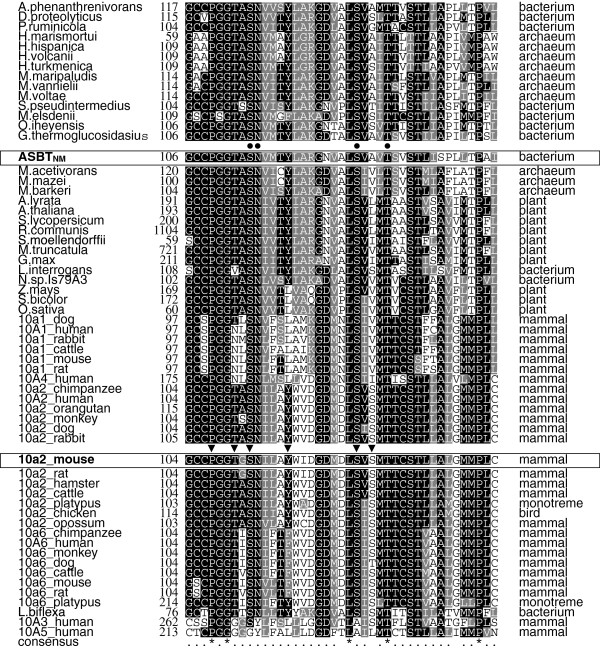
**Alignment of amino acid sequences of SLC10A family members and related proteins.** The amino acid sequences of 10 of the related genes from bacteria, archaea, and plants that yielded the highest score by a BLAST search using the amino acid sequence corresponding to Gly^104^–Pro^142^ of mouse Slc10a2 (mSlc10a2) as a query sequence and the amino acid sequence of ASBT_NM_ were compared with the amino acid sequences of SLC10A family members using the ClustalW software, and shading was rendered by the BoxShade program (http://www.ch.embnet.org/software/BOX_form.html). The alignments of the region corresponding to the query sequence are shown. The ASBT_NM_ and mSlc10a2 sequences are boxed and the residues subjected to mutational analysis in this study and residues that have been reported to bind sodium *via* their side chains [20] are indicated by arrowheads and circles, respectively.

Genes homologous to the mammalian SLC10 family are widespread in various species [25,27]. In the alignment of the deduced sequences of these genes, conserved residues are scattered throughout the entire sequences, and some of them are clustered in a region spanning approximately 40 residues corresponding to Gly^104^–Pro^142^ of SLC10A2 (Figure 1). The high-level conservation indicates that this region may play an important role in substrate interaction, conformational change necessary for function, or interaction with cellular cofactors. As mentioned above, a part of this region corresponding to EL1 has been proposed to function as a dynamic re-entrant loop, but the importance of this region has not yet been fully elucidated. To determine the importance of this region, we previously performed mutational analysis of the uncharged polar residues in the distal one-third (Thr^130^–Pro^142^) of the highly conserved region of mouse Slc10a2 (mSlc10a2) and identified residues that may be involved in substrate recognition, transport activity, and cellular localization [28]. In this study, we focused on the uncharged polar residues (Thr^110^, Ser^113^, Tyr^117^, Ser^126^, and Ser^128^) and proline (Pro^107^) in the remaining part of the highly conserved region. The hydroxyl group of the side chains could form hydrogen bonds with the substrate and/or other residues and contribute to the transport process and/or formation of higher-order structures, and proline is expected to contribute to the formation of higher-order structure.

To determine the involvement of these residues in substrate recognition, transport, and intracellular sorting and/or stability of mSlc10a2, taurocholic acid (TCA) transport and cell surface localization were analyzed.

## Results

**Figure 2:**
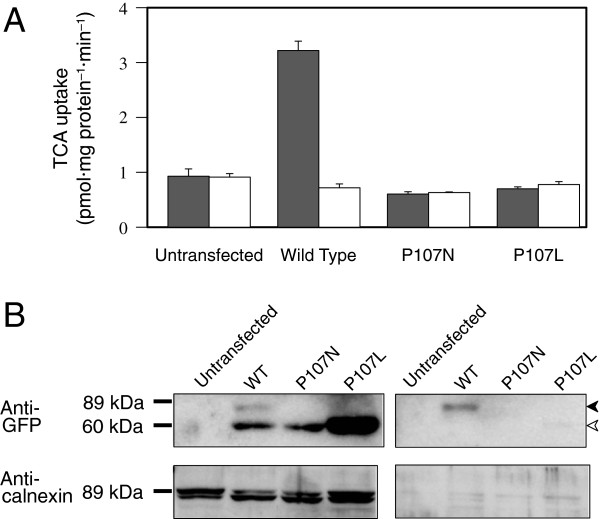
**Taurocholic acid (TCA) uptake and cellular localization of wild-type and Pro**^**107 **^**mutant Slc10a2 in stably transfected LLC**-**PK1 cells.A**, Untransfected or stably transfected LLC-PK_1_ cells were incubated with 0.1 μM [G-^3^H]TCA (44.03 GBq/mmol; Perkin-Elmer) in the presence of 100 mM NaCl (shaded columns) or 100 mM choline chloride (open columns) at 37°C for 1 min. Each column and error bar represents the mean and standard error (SE) of 3 independent experiments, respectively. **B**, Whole cell lysates (left panel) and plasma membrane fractions of untransfected or stably transfected LLC-PK_1_ cells were divided into 2 equal parts and subjected to 12.5% SDS-polyacrylamide gel electrophoresis (PAGE). Western blotting was performed using anti-green fluorescent protein (GFP) and anti-calnexin (endoplasmic reticulum marker) antibodies. Filled and open arrowheads indicate fully glycosylated and unglycosylated forms, respectively. Numbers denote molecular weight markers.

Wild-type mSlc10a2 and Pro^107^ mutants (P107N and P107L) were stably expressed as enhanced green fluorescent protein (EGFP) fusion proteins in LLC-PK_1_ cells, and TCA uptake and cellular localization were analyzed. Cells expressing wild-type Slc10a2 exhibited Na^+^-dependent uptake of TCA, but this transport activity was completely abolished in both P107N- and P107L-expressing cells (Figure 2A). Western blot analysis of whole cell lysates revealed that wild-type and mutant mSlc10a2 were abundantly expressed as 55-kDa bands, and an additional band with a lower migration rate at approximately 75 kDa was observed only in the wild type (Figure 2B, left panel). Because the latter band is considered to represent a fully glycosylated protein that is localized to the plasma membrane, we concluded that the mutant Slc10a2 proteins were likely detained in the endoplasmic reticulum (ER) and did not reach the cell surface. This was confirmed by analyzing plasma membrane proteins (Figure 2B, right panel). The glycosylated form was detected only in the wild-type plasma membrane fraction.

**Figure 3:**
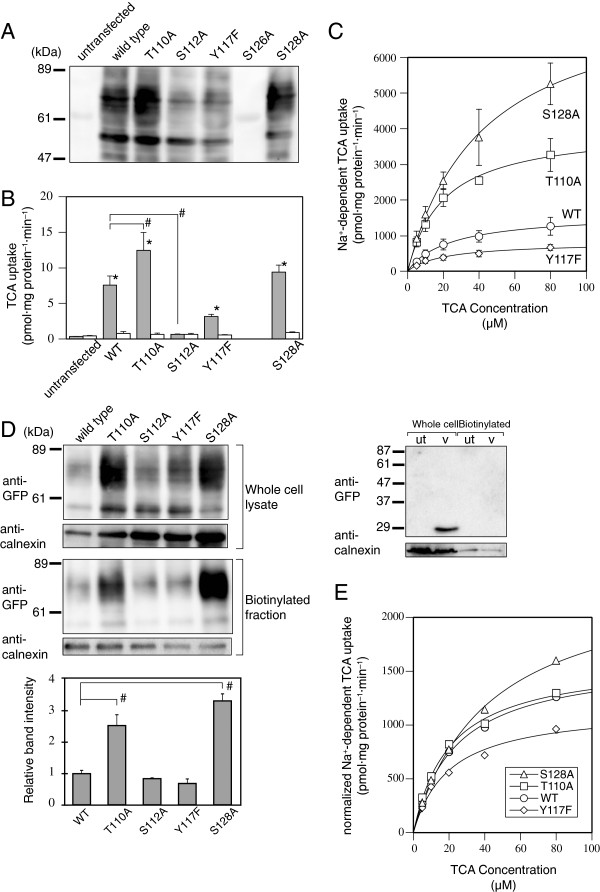
**Transient expression of wild-type and uncharged polar residue mutants of mSlc10a2 and cell surface biotinylation. A**, Twenty micrograms of whole cell lysates of COS-7 cells transiently expressing wild-type or mutant mSlc10a2 fused with EGFP were subjected to 7.5% SDS-PAGE and western blotting was performed using anti-GFP antibody. Consistency of protein loading was confirmed by Ponceau S staining of the blotted PVDF membrane (not shown). **B**, COS-7 cells transiently expressing wild-type or mutant mSlc10a2 were incubated with 0.1 μM^3^H-TCA (185 GBq/mmol; Perkin-Elmer) for 5 min in the presence of 100 mM NaCl (shaded columns) or 100 mM choline chloride (open columns). Each column and error bar represents the mean and SE of 3 independent experiments, respectively. *, Uptake in the presence of NaCl is significantly higher than that in the presence of choline chloride (*p* < 0.05); #, Na^+^-dependent uptake is significantly different from that of wild-type Slc10a2 (*p* < 0.05). **C**, Na^+^-dependent uptake of TCA was calculated by subtracting the uptake in the presence of 100 mM choline chloride from that in the presence of 100 mM NaCl. Data represent the mean and SE of 3 independent experiments. **D**, Left panel, Whole cell lysates and biotinylated fractions of COS-7 cells transiently expressing wild-type or mutant mSlc10a2 were subjected to western blot analysis. The image is representative of 3 independent experiments. The graph shows the densitometry of bands ranging from 65–90 kDa expressed as the mean and SE of 3 independent experiments. Right panel, Western blot analysis of whole cell lysates and biotinylated fractions of untransfected (ut) and empty vector-transfected (v) COS-7 cells. Because the migration rate of EGFP (26.9 kDa) encoded by empty vector is smaller than that of EGFP-fused mSlc10a2, proteins were resolved on a higher concentration (12.5%) of SDS-polyacrylamide gel. **E**, Na^+^-dependent TCA uptake by wild-type and mutant mSlc10a2 were normalized to their cell surface expression.

Next, we examined the transient expression of wild-type mSlc10a2 and uncharged polar residue mutants (T110A, S112A, Y117F, S126A, and S128A) in COS-7 cells by western blot analysis (Figure 3A). Wild-type mSlc10a2 and all the mutants except S126A were expressed as 55-kDa proteins. A broad band ranging from 60–80 kDa, the fully glycosylated form, was also detected in each lane.

To evaluate the ability of the mutants to transport bile acids, TCA uptake by the wild-type and mutant mSlc10a2 was compared (Figure 3B). Because cellular expression of S126A was not detected, this mutant was omitted from further analysis. The S128A mutant exhibited uptake levels comparable to that of wild type, and TCA uptake by the T110A mutant was significantly higher than that of wild type. TCA uptake by the Y117F mutant was approximately half that by the wild type, but the difference was not statistically significant. S112A did not exhibit Na^+^-dependent TCA uptake.

The kinetics of TCA transport by the wild type and the 3 functional mutants were examined (Figure 3C and Table 1). The *V*_max_ of Y117F was approximately half that of the wild type, and those of T110A and S128A were 2.5- and 5-fold higher than that of the wild type, respectively; interestingly, the *K*_m_ values of the 3 mutants were comparable to that of the wild type, suggesting that the differences in the apparent TCA transport activities of the polar residue mutants were not due to altered affinity for TCA.

**Table 1:** Kinetic values for taurocholic acid transport by wild-type and polar-residue mutant mouse Slc10a2 proteins

	***K***_**m **_**(μM)**	***V***_**max **_**(pmol·mg protein**^**–1**^**·min**^**–1**^**)**
Wild type	24.0 (2.0)	1620 (50)
T110A	20.2 (2.3)	4010 (170)
Y117F	20.7 (4.1)	806 (61)
S128A	42.0 (3.7)	7930 (340)

Data are expressed as mean (standard error).

The cellular localization of the expressed transporters was investigated by cell surface biotinylation (Figure 3D). The fully glycosylated form was predominantly detected in the biotinylated fraction. Membrane expression of Y117F was similar to that of wild type, and the membrane expression of T110A and S128A was significantly higher than that of wild type, suggesting that removal of the polar hydroxyl group from Thr^110^ and Ser^128^ improved membrane sorting and/or stability of mSlc10a2. Although S112A was a loss-of-function mutation, membrane expression of the mutant was clearly detected, indicating that Ser^112^ is critical for the activity of mSlc10a2.

To compare the activities of the mutant transporters, the Na^+^-dependent TCA uptake by wild-type and mutant mSlc10a2 was normalized to the cell surface expression of the corresponding proteins (Figure 3E). The normalized *V*_max_ of the T110A mutant (1600 [70] pmol·mg protein^–1^·min^–1^) was similar to that of the wild type (1620 [50] pmol·mg protein^–1^·min^–1^), indicating that the difference in the apparent TCA transport activity of this mutant was mainly due to the different level of its expression at the cell surface. By contrast, the apparently higher activity of S128A could not be explained by abundant membrane expression because the normalized *V*_max_ of the S128A mutant (2410 [100] pmol·mg protein^–1^·min^–1^) was remarkably higher than that of wild type. The normalized *V*_max_ value of the Y117F mutant (1170 [90] pmol·mg protein^–1^·min^–1^) was lower than that of wild type.

**Figure 4:**
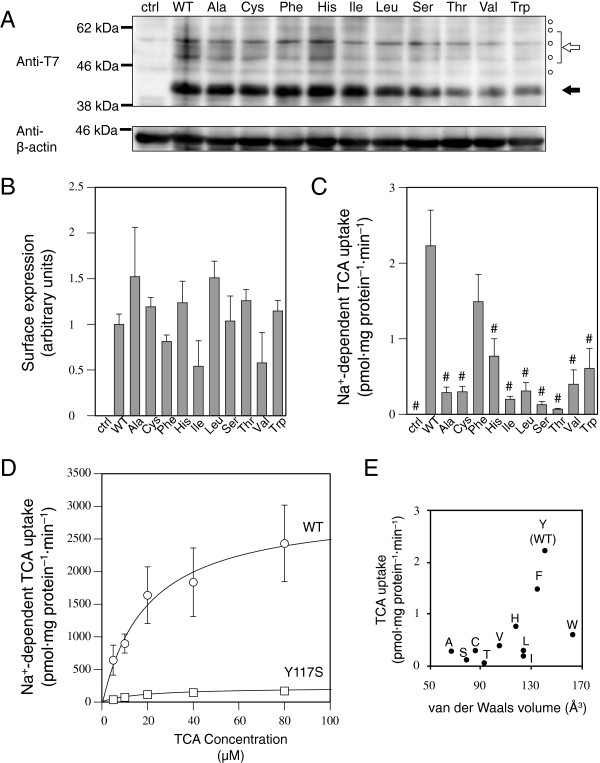
**Expression and transport activities of Tyr**^**117 **^**mutants. A**, COS-7 cells were transiently transfected with expression vectors for T7-tagged wild-type or mutant mSlc10a2. Whole cell lysates were examined by western blotting using anti-T7 antibody (upper). Whole cell lysate of untransfected cells was loaded as a control (ctrl). Filled and open arrows indicate core-glycosylated and fully glycosylated forms of T7-mSlc10a2, respectively. T7-tag constructs yield lower levels of the fully glycosylated form compared to EGFP-fusion constructs. Circles indicate apparently nonspecific bands. The membrane was stripped and reprobed with anti-β-actin antibody (lower). **B**, Cell surface expression was measured by surface enzyme-linked immunosorbent assay. Data are expressed as mean and SE of a sextuplicate experiment. **C**, COS-7 cells transiently expressing wild-type or mutant mSlc10a2 were incubated with 0.02 μM ^3^H-TCA (370 GBq/mmol; American Radiolabeled Chemicals) for 5 min in the presence of 100 mM NaCl or 100 mM choline chloride, and Na^+^-dependent uptake was calculated by subtracting the uptake in the presence of 100 mM choline chloride from that in the presence of 100 mM NaCl. Na^+^-dependent uptake data are expressed as mean and SE of 3 independent experiments. #, Na^+^-dependent uptake is significantly different from that of wild-type Slc10a2 (*p* < 0.05). **D**, Kinetic analysis of Na^+^-dependent TCA transport by wild-type and Y117S. Data are expressed as the mean and SE of 3 independent experiments. Circles, wild type; squares, Y117S. **E**, Na^+^-dependent TCA uptake by the wild-type mSlc10a2 and Tyr^117^ mutants are plotted against the van der Waals volume of the residue at position 117.

**Figure 5:**
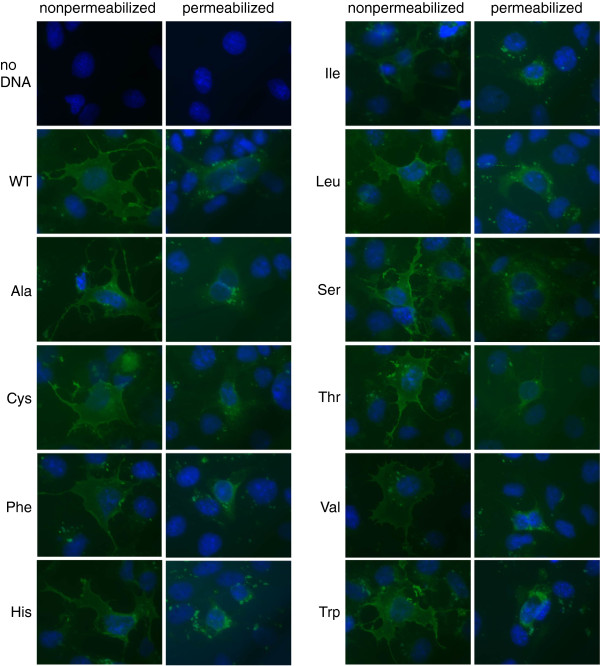
**Immunofluorescence microscopy of wild-type and Tyr117 mutant mSlc10a2.** COS-7 cells transiently expressing T7-tagged mSlc10a2 proteins were incubated with anti-T7 antibody under nonpermeabilized (left panel) or permeabilized (right panel) conditions, followed by fluorescein-conjugated secondary antibody. The merged image of mSlc10a2 immunofluorescence (green) and nuclear staining (blue) is shown.

The Y117C mutation in human SLC10A2 has been reported to be a loss-of-function mutation due to its impaired membrane expression [23]. This is in striking contrast to our finding that the Y117F mutant of mSlc10a2 was properly expressed on the plasma membrane and that transport activity was preserved even though its apparent activity was lower. Because the Tyr to Phe mutation used in the present study removed only the polar hydroxyl group from the benzene ring of the side chain, we infer that what is important at this position is not polarity but the bulkiness required for higher-order structure or stability of SLC10A2/Slc10a2. The complete conservation of bulky residues, i.e., Tyr, Phe, or Leu, at this position in the related genes (Figure 1) supports this view. Therefore, we next examined the cell surface expression and transport activities of mSlc10a2 mutants in which various residues were substituted for Tyr^117^. For this purpose, a T7 tag was attached at the N-terminus of mSlc10a2, which is exposed on the cell surface. Western blot analysis revealed that the wild-type and Tyr^117^ mutants were expressed as 40-kDa bands; additional bands with lower migration rates, ranging from 50–60 kDa, were also detected in each lane, indicating that all the Tyr^117^ mutants as well as the wild type were properly expressed on the plasma membrane (Figure 4A). Cell surface expression was confirmed with immunofluorescence. Under nonpermeabilized conditions, the plasma membrane was predominantly stained, whereas additional intracellular compartments were also stained under permeabilized conditions (Figure 5). Cell surface expression levels were examined by surface enzyme-linked immunosorbent assay. The cell-surface expression of the Tyr^117^ mutants was not significantly lower than that of wild type (Figure 4B). Na^+^-dependent TCA uptake by all the Tyr^117^ mutants, except Y117F, was significantly lower than that of wild type (Figure 4C). Next, we analyzed the kinetic properties of the Y117S mutant, which exhibited remarkably reduced TCA uptake compared to that of wild type. The *K*_m_ for Na^+^-dependent TCA uptake by the Y117S mutant (19.1 [2.4] μM) was similar to that of wild type (19.8 [4.7] μM), whereas the *V*_max_ of Y117S (216 [10] pmol·mg protein^–1^·min^–1^) was remarkably lower than that of wild type (2965 [263] pmol·mg protein^–1^·min^–1^) (Figure 4D). These results indicate that the apparently decreased activities of the Tyr^117^ mutants are probably due to their decreased molecular activities.

## Discussion

Optimal function of SLC10A2 is required for bile acid reabsorption in the ileum. Impairment of this function not only affects cholesterol homeostasis but may also increase the possibility of colorectal tumorigenesis due to increased flow of bile acids into the large intestine. Indeed, prevention of bile acid reabsorption by surgical removal of the ileum increased colonic tumorigenesis in rats fed deoxycholic acid [29]. The C to T polymorphism at codon 169 of the human *SLC10A2* gene is associated with colorectal adenomas, indicating the role of bile acids in the etiology of this disease [30]. A genetic polymorphism associated with primary bile acid malabsorption (PBAM) or idiopathic intestinal bile acid malabsorption (IBAM) has been identified, and mutations that abolish transport function (L243P and T262M) and a haplotype block linked to reduced expression have been reported for human SLC10A2 [31,32]. This polymorphism has not been mapped in the highly conserved region, and the importance of the cluster of conserved residues has not yet been fully clarified. Toward the end of the study presented here, the crystal structure of ASBT_NM_ was reported, and some of the residues were indicated to form a part of the Na^+^-binding pocket [20].

We have previously reported the importance of Pro^142^, which is located at the distal end of the highly conserved region [28]. Substitution of Pro^142^ with Val completely impaired cell surface localization of mSlc10a2. This is consistent with our results from the mutational analysis of Pro^107^ showing that Asn or Leu substitution for Pro^107^ impaired cell surface expression of mSlc10a2, resulting in the loss of transport activity. In the 9-TM model, Pro^107^ is located in the middle of TM3. Proline acts as a “helix breaker” due to its inability to form hydrogen bonds with neighboring residues; therefore, TM3 would be bent at Pro^107^, forming hydrophobic and amphipathic half helices. Indeed, the crystal structure of ASBT_NM_ suggests that the helix that contains Pro^107^ is broken precisely at this residue [20]. In the 7-TM model, Pro^107^ is located in the EL between TM2 and TM3. In the ER, immediately after the synthesis of the nascent protein, this loop faces the lumen. Substitution of Pro^107^ with Asn or Leu may have introduced an interaction of this loop with other ELs or ER factors, resulting in detention of the mutant Slc10a2. In either case, failure of the Pro^107^ mutants to localize to the cell surface suggests that the peculiar nature of proline, an imino acid, and not the hydrophobicity or bulkiness of the side chain is important for intracellular sorting. Given that proline at this position is highly conserved in the related proteins, it is expected to be crucial for function through correct secondary structure formation and cellular localization.

Ser^112^ was considered indispensable for the synthesis or stability of human SLC10A2 because replacement of Ser^112^ with Cys completely abolished expression [23]. In this study, however, Ala substitution of Ser^112^ did not impair membrane expression of mSlc10a2, whereas TCA transport activity was completely lost. This suggests that Ser^112^ is not critical for mSlc10a2 expression but is essential for TCA transport activity. Based on the crystal structure of ASBT_NM_, Ser^112^ of mSlc10a2 is thought to bind Na^+^ with its side chain [20]. Ser is conserved at this position in all the related proteins except SLC10A5 (Figure 1), suggesting that this residue is also critical in all the other members.

Expression of the S126A mutant was undetectable even in whole cell lysates. This is consistent with a previous report showing the failure of expression of the human SLC10A2 S126C mutant [23]. This residue has been suggested to bind Na^+^[20], and it is likely that Ser^126^ plays an important role in the other members as well, given that this residue is conserved in all the genes except those encoding SLC10A3 and SLC10A5.

Because Thr^110^ and Ser^128^ are not conserved in the related proteins, particularly in the Na^+^-dependent bile acid transporter SLC10A1 (also designated NTCP or BSBT), which is expressed on the sinusoidal membrane of hepatocytes, it is unlikely that these residues are involved in the interaction with bile acids. The apparently higher membrane expression of the T110A and S128A mutants suggests that these residues may be involved in the negative regulation of stability or intracellular sorting *via* formation of higher-order structures or interaction with cellular cofactors.

The Phe substitution for Tyr^117^, which removes a polar hydroxyl group, reduced the transport activity of mSlc10a2; however, substrate affinity was not affected. This result was inconsistent with the previous finding that the Y117C mutation completely impaired membrane expression of human SLC10A2. To resolve this discrepancy, we replaced Tyr^117^ of mSlc10a2 with various amino acids and examined cellular localization and transport activities. None of the residues substituted for Tyr^117^ affected cell surface expression of mSlc10a2; however, the apparent transport activities were reduced. The reason for the contradiction between our results and the results of the Y117C mutation in human SLC10A2 is not clear, but a difference in the amino acid sequence context between mSlc10a2 and the human counterpart or undefined differences in experimental conditions may have affected the results. The relationship between the apparent activities and physicochemical properties of the residues were analyzed. Hydrophobicity and polarity did not correlate with activity (data not shown), but a weak correlation was observed between the van der Waals volumes of the residues and the activities of the mutants (Figure 4E). However, the correlation was not statistically significant (*p* = 0.0705), because the Y117W mutant with the bulkiest side chain exhibited moderate activity, and substitution with Ile and Leu, which have relatively bulky side chains, elicited low transport activities, suggesting that the volume of the side chain as well as its shape are important at this position. Although the crystal structure of ASBT_NM_ indicated that Tyr^117^ does not directly interact with Na^+^ or taurocholate, it is obvious that this residue is important for the molecular activity of the transporter. Tyr^117^ may be involved in conformational changes during TCA transport.

## Conclusions

In this study, residues critical for transport activity, expression, and stability and/or intracellular trafficking, but not substrate recognition, were identified within the proximal two-thirds of the highly conserved region. Functionally important residues are clustered in the highly conserved region. Due to the specific and high transport capacity of SLC10A2 in the ileum, bioavailability of drugs may be enhanced by designing them as bile acid-conjugated prodrugs. The information on functionally critical residues will contribute to the design of prodrugs for efficient drug delivery and SLC10A2 inhibitors for treatment of hypercholesterolemia.

## Methods

### Materials

[G-^3^H]TCA was purchased from Perkin-Elmer (Waltham, MA) and American Radiolabeled Chemicals (St. Louis, MO). Unlabeled sodium taurocholate was purchased from Nacalai Tesque (Kyoto, Japan). The Cell Surface Protein Isolation Kit, which included sulfosuccinimidyl 2-(biotinamido)ethyl-1,3-dithiopropionate (sulfo-NHS-SS-biotin), was purchased from Pierce (Rockford, IL). Streptavidin agarose was purchased from Merck (Darmstadt, Germany). The anti-green fluorescent protein (GFP) monoclonal antibody was purchased from Nacalai Tesque. The anti-T7 tag monoclonal antibody was purchased from Merck. The anti-calnexin rabbit polyclonal antibody was from Novus Biologicals (Littleton, CO). Secondary antibodies (horseradish peroxidase (HRP)-conjugated anti-mouse, anti-rat, and anti-rabbit IgG) were purchased from Nacalai Tesque. Fluorescein-labeled anti-mouse secondary antibody was purchased from Kirkegaard & Perry Laboratories (Gaithersburg, MD). The cloning vector pUC119 was purchased from Takara Bio (Shiga, Japan). The mammalian expression vectors pEGFP-N1 and pZeoSV2(+) were purchased from Clontech (Shiga, Japan) and Invitrogen (Tokyo, Japan), respectively. Mutagenic oligonucleotide primers for site-directed mutagenesis were custom synthesized and purchased from Invitrogen. Site-directed mutagenesis of Pro^107^ and uncharged polar residues was performed using the Quickchange II Site-Directed Mutagenesis Kit purchased from Stratagene (La Jolla, CA). Site-directed mutagenesis of Tyr^117^ was performed using the PrimeSTAR Mutagenesis Basal Kit purchased from Takara Bio.

### Cell culture

The simian kidney fibroblast cell line COS-7 was grown in Dulbecco’s modified Eagle’s medium supplemented with 10% fetal bovine serum (FBS), 4 mM l-Gln, and 0.1 mg/mL kanamycin sulfate. The porcine kidney cell line LLC-PK_1_ was grown in M199 medium supplemented with 10% FBS and 0.1 mg/mL kanamycin. Cells were cultured at 37°C in a humidified atmosphere of 5% CO_2_.

### Construction of expression vectors

The mSlc10a2 cDNA encoding the full-length transporter (molecular weight, 38 kDa) was cloned in our laboratory [33]. Wild-type or mutant mSlc10a2 was expressed as a fusion protein with enhanced green fluorescent protein (EGFP) or a T7 tag. The expression vectors for EGFP-fused mSlc10a2 were constructed as previously reported [28]. To construct the expression vector for T7-tagged mSlc10a2, an *Eco*RI recognition site was introduced at the start codon by polymerase chain reaction (PCR) using the 5^′^ primer 5^′^-CGAATTCAGATGGATAACTCCTCTGTCTG-3^′^, in which the underlined portion represents the start codon, and the 3^′^ primer 5^′^-GAAGGATCCCCATGGTCTCTTTATATGTCC-3^′^ corresponding to nucleotides 178–207 of the coding region in which a *Bam*HI site was introduced without affecting the amino acid sequence. The amplified fragment was cloned into pUC119 and sequenced to confirm the absence of PCR-derived mutations. The *Eco*RI-*Bam*HI fragment obtained from the amplified clone and the remaining part of the coding region were cloned into pZeoSV2(+), and a double-stranded synthetic oligonucleotide encoding the T7 tag next to the start codon (top strand, 5^′^-CTAGCATGGGGATGGCTAGCATGACTGGTGGACAACAGATGGGTGG-3^′^; bottom strand, 5^′^-AATTCCACCCATCTGTTGTCCACCAGTCATGCTAGCCATCCCCATG-3^′^, where the underlined portions represent the T7 tag) was inserted at the *Nhe*I and *Eco*RI sites to construct an expression vector designated pSV40-T7-mSLC10A2.

### Site-directed mutagenesis

An *Eco*RI-*Bam*HI restriction fragment corresponding to nucleotides 1–589 of the coding region of mSlc10a2 cDNA was cloned into pUC119, and site-directed mutagenesis was performed according to the manufacturer’s instructions. Mutagenesis of Pro^107^ was performed using synthetic double-stranded primers (sense primer, 5^′^-GCTAATTATGGGTTGCTGCNNNGGAGGAACTGGCTCC-3^′^; and antisense primer, 5^′^-GGAGCCAGTTCCTCCNNNGCAGCAACCCATAATTAGC-3^′^; where the underlined portions represent the Pro^107^ codon, and N indicates a randomized nucleotide). Following nucleotide sequence analysis, 2 clones with Asn and Leu substitutions for Pro^107^ were obtained. Mutagenesis of uncharged polar residues was performed using synthetic double-stranded primers (T110A sense primer, 5^′^-GCTGCCCTGGAGGAGCTGGCTCCAATATCC-3^′^, and antisense primer, 5^′^-GGATATTGGAGCCAGCTCCTCCAGGGCAGC-3^′^; S112A sense primer, 5^′^-CTGGAGGAACTGGCGCCAATATCCTGGCC-3^′^, and antisense primer, 5^′^-GGCCAGGATATTGGCGCCAGTTCCTCCAG-3^′^; Y117F sense primer, 5^′^-GCTCCAATATCCTGGCCTTTTGGATAGATGGCG-3^′^, and antisense primer, 5^′^-CGCCATCTATCCAAAAGGCCAGGATATTGGAGC-3^′^; S126A sense primer, 5^′^-GGCGACATGGACCTCGCTGTTAGCATGACCACTTGC-3^′^, and antisense primer, 5^′^-GCAAGTGGTCATGCTAACAGCGAGGTCCATGTCGCC-3^′^; S128A sense primer, 5^′^-CATGGACCTCAGTGTTGCCATGACCACTTGCTCCAC-3^′^, and antisense primer, 5^′^-GTGGAGCAAGTGGTCATGGCAACACTGAGGTCCATG-3^′^; where the underlined portions represent the target codons). Mutagenesis of Tyr^117^ was performed using synthetic double-stranded primers (sense primer, 5^′^-CTGGCCTATTGGATAGATGGCGACAT-3^′^; and antisense primer, 5^′^-TATCCAATAGGCCAGGATATTGGAGCC-3^′^; the underlined portions represent the Tyr^117^ codon of wild-type mSlc10a2, and this codon was replaced with the following sequences: Y117A, GCT; Y117C, TGT; Y117H, CAT; Y117I, ATT; Y117L, CTT; Y117S, TCT; Y117T, ACT; Y117V, GTT; Y117W, TGG). All the mutations were verified by DNA sequencing. The corresponding segment of the expression vector was replaced with the restriction fragment containing the expected mutation.

### Transient expression and TCA uptake

On the day before transfection, 2.4 × 10^5^ COS-7 cells were seeded in a 3.5-cm dish. The cells were transfected with appropriate expression vectors using Lipofectamine and Plus reagent (both purchased from Invitrogen), according to the manufacturer’s instructions. Two days after transfection, uptake of ^3^H-TCA was measured as previously described with slight modifications [34]. The cells were washed twice with a wash buffer (10 mM Tris-HCl, pH 7.4, 200 mM mannitol), and were then covered with 1 mL of uptake buffer (10 mM Tris-HCl, pH 7.4, 100 mM NaCl or choline chloride, 3 mM K_2_HPO_4_) containing the indicated concentration of ^3^H-TCA and incubated at 37°C. The reaction was stopped by washing cells twice with 1 mL of ice-cold wash buffer, and cells were lysed in 1 mL of 0.2 M NaOH. Cell-associated radioactivity was measured using a liquid scintillation counter (Perkin-Elmer) and normalized to the total protein content determined using the Bradford method with bovine serum albumin as a concentration standard. For quantitative transport analysis, TCA was used at a concentration range much lower than the critical micellar concentration.

Apparent *K*_m_ and *V*_max_ values for TCA uptake were determined by measuring the initial rates of uptake at various concentrations of taurocholate. The TCA concentration was adjusted by adding unlabeled TCA. The data were fitted to the Michaelis-Menten equation by nonlinear regression using the KaleidaGraph 4.0 software (Synergy Software, Reading, PA).

### Cell surface biotinylation

Biotinylation of cell surface proteins was performed using the Cell Surface Protein Isolation Kit according to the manufacturer’s instructions with modifications. COS-7 cells were transfected with pZmISBT-EGFP2, as described above, and incubated for 2 days. The cells were washed twice with ice-cold phosphate-buffered saline (PBS; 0.1 M sodium phosphate, 0.15 M NaCl, pH 7.2) and then covered with a membrane-impermeable biotinylating reagent (2.0 mL of 1.5 mg/mL sulfo-NHS-SS-biotin dissolved in ice-cold PBS) at 4°C for 30 min with constant agitation. The reaction was stopped by adding 100 μL of quenching solution (provided as a component of the kit) and washed with Tris-buffered saline (0.025 M Tris-HCl, 0.15 M NaCl, pH 7.2). The cells were then covered with 700 μL of lysis buffer (150 mM NaCl, 1 mM EDTA, 0.1% SDS, 1% Triton X-100, 10 mM Tris-HCl, pH 7.4) supplemented with protease inhibitors (2 mM phenylmethylsufonyl fluoride, 0.022 trypsin inhibitor units/mL aprotinin, 5 μg/mL leupeptin, 0.1 μg/mL pepstatin) at 4°C for 1 h with constant agitation. The lysate was centrifuged at 13,000 rpm at 4°C for 10 min, and 600 μL of the supernatant was collected. The supernatant was incubated with 40 μL of 50% (vol/vol) streptavidin-agarose beads for 1 h with constant agitation. The mixture was centrifuged at 13,000 rpm for 2 min, and the supernatant was discarded. The beads were washed 4 times with ice-cold lysis buffer and suspended in 20 μL of 2 × SDS sample buffer (30% glycerol, 1% SDS, 0.093 g/mL DTT, 0.12 mg/mL bromophenol blue, 0.35 M Tris-HCl, pH 6.8). Ten microliters of the samples was subjected to 7.5% SDS-polyacrylamide gel electrophoresis, and western blotting was performed using anti-GFP antibody. The blots were then immersed in 0.2 M NaOH for 5 min to remove antibodies, and washed with distilled water for 5 min. The blots were reprobed with anti-calnexin antibody. The relative intensities of the protein bands were analyzed using Image J software (http://rsb.info.nih.gov/ij/).

### Plasma membrane fractionation

The plasma membrane fraction was extracted from cells cultured in a 10-cm dish using the Plasma Membrane Protein Extraction Kit (Biovision, Milpitas, CA), according to the manufacturer’s instructions.

### Immunofluorescence microscopy and surface ELISA

COS-7 cells were transfected with pSV40-T7-mSLC10A2. Two days after transfection, the cells were washed 3 times with PBS and incubated with 4% paraformaldehyde dissolved in PBS for 20 min at room temperature. The cells were then washed 3 times with PBS. For immunofluorescence under permeabilized conditions, the cells were incubated with 0.2% Triton X-100 for 20 min and blocked with 1% BSA in PBS for 30 min at room temperature. For nonpermeabilized conditions, incubation with Triton X-100 was omitted. The cells were incubated with anti-T7 tag monoclonal antibody at 1/2000 dilution for 30 min at room temperature. The cells were washed 3 times with PBS and incubated with fluorescein-conjugated anti-mouse antibody at 1/500 dilution for 1 h in the dark. The cells were stained with 10 μg/mL Hoechst 33342 for 5 min and washed 3 times with PBS in the dark. The cells were examined under a microscope (Axio Imager M1; Carl Zeiss, Tokyo, Japan), and images were captured using a digital camera (AxioCam MRm; Carl Zeiss). For surface ELISA, the cells were incubated with HRP-conjugated anti-T7 tag monoclonal antibody at 1/2000 dilution for 1 h at room temperature. The cells were washed 3 times with PBS, and incubated with SuperSignal ELISA Femto Maximum Sensitivity Substrate (Pierce) for 1 min at room temperature. Luminescence was measured using a plate reader (2030 ARVO X3; PerkinElmer).

### Alignment of amino acid sequences

The nucleotide sequences of the related genes were retrieved by a BLAST search (tblastn program provided at http://blast.ncbi.nlm.nih.gov/) using the amino acid sequence “GCCPGGTGSNILAYWIDGDMDLSVSMTTCSTLLALGMMP” corresponding to Gly^104^–Pro^142^ of mouse Slc10a2 (mSlc10a2) as a query sequence. The deduced amino acid sequences of 10 of the identified genes from bacteria, archaea, and plants that yielded the highest score and the amino acid sequence of ASBT_NM_ were compared with the amino acid sequences of SLC10A family members using the ClustalW software. The amino acid sequences were deduced from GenBank nucleotide sequences with the following accession numbers: *Arthrobacter phenanthrenivorans*, [CP002379]; *Deinococcus proteolyticus*, [CP002537]; *Prevotella ruminicola*, [CP002006]; *Haloarcula marismortui*, [AY596296]; *Haloarcula hispanica*, [CP002923]; *Haloferax volcanii*, [CP001956]; *Haloterrigena turkmenica*, [CP001860]; *Methanococcus maripaludis*, [CP000609]; *Methanococcus vannielii*, [CP000742]; *Methanococcus voltae*, [CP002057]; *Staphylococcus pseudintermedius*, [CP002439]; *Megasphaera elsdenii*, [HE576794]; *Oceanobacillus iheyensis*, [BA000028]; *Geobacillus thermoglucosidasius*, [CP002835]; *Methanosarcina acetivorans*, [AE010299]; *Methanosarcina mazei*, [AE008384]; *Methanosarcina barkeri*, [CP000099]; *Arabidopsis lyrata*, [XM_002889153]; *Arabidopsis thaliana*, [BX816582]; *Solanum lycopersicum*, [AK320352]; *Ricinus communis*, [XM_002531199]; *Selaginella moellendorffii*, [FJ51633]; *Medicago truncatula*, [XM_003638354]; *Glycine max*, [XM_003543135]; *Leptospira interrogans*, [AE010301]; *Nitrosomonas* sp. Is79A3, [CP002876]; *Zea mays*, [NM_001158879]; *Sorghum bicolor*, [XM_002442850]; *Oryza sativa*, [NM_001189880]; *Leptospira biflexa*, [CP000786]; *SLC10A1*/*Slc10a1*, dog, [XM_537494]; human, [NM_003049]; rabbit, [NM_001082768]; cattle, [BC105471]; mouse, [NM_011387]; rat, [NM_017047]; *SLC10A2*/*Slc10a2*, chimpanzee, [XM_522716]; human, [NM_000452]; orangutan, [NM_001131608]; *Macaca mulatta* (rhesus monkey), [XM_001095212]; dog, [NM_001002968]; rabbit, [NM_001082764]; mouse, [NM_011388]; rat, [NM_017222]; hamster, [NM_001246820]; cattle, [XM_604179]; platypus, [XM_001513315], chicken, [XM_425589]; opossum, [XM_001376304]; human *SLC10A3*, [NM_019848]; *SLC10A4*, [NM_152679]; *SLC10A5*, [NM_001010893]; *SLC10A6*/*Slc10a6*, chimpanzee, [XM_526626]; human, [NM_197965]; monkey, [XM_001092284]; dog, [XM_846210]; cattle, [NM_001081738]; mouse, [NM_029415]; rat, [NM_198049]; platypus, [XM_001515822]. The amino acid sequence of ASBT_NM_ was obtained from UniProt [Q9K0A9].

### Statistical analysis

TCA uptake and biotinylation results were analyzed by Dunnett’s test and Student’s t-test using the JMP software (SAS Institute, Tokyo, Japan).

## Abbreviations

EGFP: Enhanced green fluorescent protein; EL: Extracellular loop; mSlc10a2: Mouse solute carrier family 10 member 2; Sulfo-NHS-SS-biotin: Sulfosuccinimidyl 2-(biotinamido)ethyl-1,3-dithiopropionate; TCA: Taurocholic acid; TM: Transmembrane.

## Competing interests

The authors declare that they have no competing interests.

## Authors’ contributions

TS designed and supervised the study, performed all the experiments except those mentioned below, and drafted the manuscript. KS constructed the expression vectors for T7-tagged Tyr^117^ mutants and performed surface ELISA (Figure 4B) and fluorescence microscopy (Figure 5). SI performed kinetic analysis of TCA uptake by the Y117S mutant (Figure 4D). KI constructed the expression vectors for EGFP-fused uncharged polar residue mutants, measured TCA uptake by these mutants, and performed western blot analyses (Figure 3). RK was the principal supervisor. All authors approved the final manuscript.
